# Predictors of Noncoaxial Anteroposterior Deployment of Transcatheter Aortic Valve Replacement

**DOI:** 10.1016/j.shj.2026.100833

**Published:** 2026-03-10

**Authors:** Sana Shaukat, Hamza Lodhi, Mohammed Ebrahim, John Nicholas Melvan, Houman Khalili

**Affiliations:** a Department of Medicine, Florida Atlantic University Hospital, Boca Raton, Florida, USA; b Division of Cardiology, Memorial Healthcare System, Hollywood, Florida, USA

**Keywords:** Implantation technique, Noncoaxial, TAVR, Transcatheter aortic valve replacement

## Abstract

• Novel measurement: This study introduces a novel technique for measuring anteroposterior (AP) axial angle to quantify the coaxiality of valve deployment in balloon-expandable transcatheter aortic valve replacement.
• Identification of key predictors: Multivariable analysis identified predeployment noncoaxial transcatheter aortic valve replacement valve position and right anterior oblique coplanar angles as independent predictors of noncoaxial valve placement.
• AP noncoaxial deployment: Although previous research has largely examined lateral noncoaxiality, this study focuses on the predictors and implications of AP noncoaxial deployment.
• Predictive heatmap visualization: The research utilized a generalized linear model to develop an unadjusted heatmap, visualizing the probability of noncoaxial implant based on computed tomography–determined fluoroscopic coplanar angles.

As transcatheter aortic valve replacement (TAVR) expands into younger, lower-risk populations, achieving optimal initial valve deployment is critical for long-term durability and the feasibility of future valve-in-valve procedures.^1^^,^^2^ Beyond valve design and patient anatomy, implantation techniques could be a determinant of procedural success. Consequences of a noncoaxial deployment could include abnormal or inefficient valve flow dynamics, sinus washout, obstructed coronary access (in patients with borderline coronary height), difficult future valve-in-valve TAVR, paravalvular leak, and pacemaker risk due to encroachment of the membranous septum by the canted segment. This study aims to identify preprocedural and intraprocedural predictors of noncoaxial anteroposterior (AP) deployment of the balloon-expandable Sapien S3 valve.

Data were gathered by a retrospective chart review of patients who had TAVR with the Sapien S3 valve between 2019 and 2021 at Delray Medical Center, Delray Beach, FL. We devised a novel method of measuring AP axial angles (Figure 1a and b). Due to the retrospective nature of the study, lateral axial angle could not be determined in most procedures due to absence of final angiography in the coplanar view. For the remainder of this letter, AP coaxial implant or AP axial angle may be referred to as “coaxial implant” or “axial angle,” respectively. Predictors of noncoaxial deployment were identified by first performing a univariable analysis of all candidate variables, including anatomical measurements (sinus of Valsalva diameter, aortic annulus to left ventricle apex angle, and interventricular septal thickness), procedural factors (coplanar angles, intraprocedure predeployment noncoaxial TAVR valve position [PDN] [Figure 1a], and guidewire type), and valve size. Variables that met a significance threshold of *p* < 0.05 were then selected for inclusion in a final multivariable model. Independent predictors were used to construct a generalized linear model. Receiver operating characteristic analysis was used to test the model performance with discriminative ability quantified by the area under the curve (AUC). To account for potential overfitting, internal validation was performed using 300 bootstrap resamples. This was used to calculate bias-corrected estimates for the AUC and the calibration slope. Calibration was also assessed using the unreliability index (*U*). An unadjusted generalized linear model was also constructed to estimate the probability of noncoaxial implant as a function of fluoroscopic angles corresponding to the coplanar view, and predicted probabilities were visualized across a Cartesian plane using a heat map derived from model outputs.

**Figure 1:**
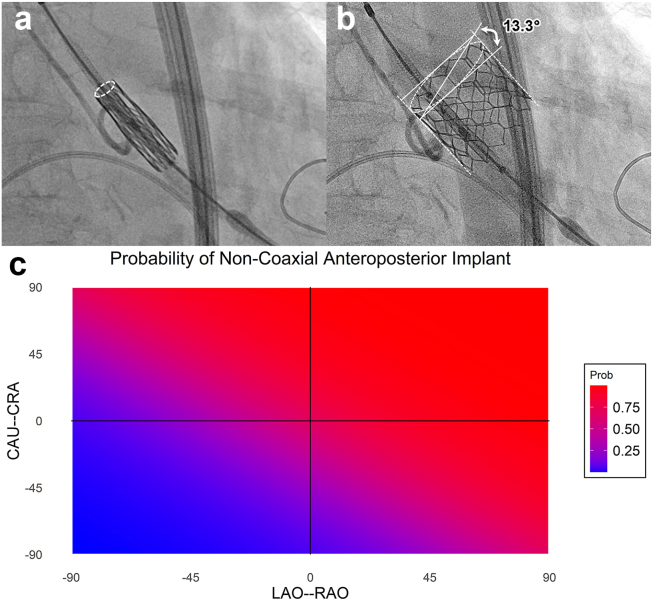
**(a)** Predeployment anteroposterior noncoaxial position depicted by failure of anterior and posterior rims to overlap, as shown by the dashed oval. **(b)** Measurement of postdeployment anteroposterior noncoaxiality. **(c)** Heat map. Abbreviation: CAU, caudal; CRA, cranial; LAO, left anterior oblique; RAO, right anterior oblique. .

This study included 184 patients. The interquartile range of the AP axial angle for the cohort was 3°-9° (median of 6°). The prevalence of ≥ 6° axial deployment was 54% (n = 100). On univariate analysis, right anterior oblique (RAO) (odds ratio [OR] 1.03, 95% CI 1.01-1.06, *p* = 0.019) and caudal coplanar angles (OR 1.03, 95% CI 1.01-1.06, *p* = 0.023) were associated with ≥ 6° axial deployment. PDN was also associated with noncoaxial implant (OR 7.70, 95% CI 3.84-16.34, *p* < 0.001). Valve size, alpha angle on computed tomography (aortic annulus to left ventricle apex angle), sinus of Valsalva diameter, interventricular septal thickness, and the type of guidewire were not associated with noncoaxial implant. On multivariable analysis, PDN (OR 6.97, 95% CI 3.39-15.19, *p* < 0.001) and RAO coplanar angle (OR 1.04, 95% CI 1.01-1.08, *p* = 0.021) remained associated with noncoaxial deployment, whereas caudal coplanar angle was not statistically significant (OR 1.03, 95% CI 1.00-1.06, *p* = 0.097).

Receiver operating characteristic analysis demonstrated that the model based on PDN and fluoroscopic coplanar angles achieved good discriminative ability for predicting noncoaxial implants, with an apparent AUC of 0.769 (95% CI 0.699-0.839). Internal validation confirmed the stability of the model, yielding an optimism-corrected AUC of 0.759. Calibration was excellent, and although the apparent calibration was near perfect (slope = 1.0, *U p*-value = 1.0), the bias-corrected calibration slope was 0.94, indicating high reliability of the predicted probabilities with minimal evidence of overfitting. Lastly, Figure 1c shows an unadjusted heat map of the probability of noncoaxial implant based on computed tomography–determined coplanar angle.

Several limitations warrant consideration. First, the study is limited by its retrospective design. Second, although the AP axial measurement is a novel proposed metric, formal interobserver variability testing is required to standardize its clinical use. Finally, the relatively small sample size precluded an assessment of the correlation between the AP axial angle ≥ 6° and acute hemodynamic or clinical outcomes (e.g., paravalvular leak or gradients) or long-term valve academic research consortium-3 safety endpoints. However, a prior study demonstrated that noncoaxial deployment is associated with lower 30-day safety (as defined by the valve academic research consortium-3 criteria) and reduced bioprosthetic valve function at 1 year.^3^ In contrast to the previous study that examined lateral noncoaxial deployment, our research concentrated on AP noncoaxial deployment. Furthermore, the influence of fluoroscopic coplanar angles—a key predictor identified in our study—was not assessed in the previous work. Mitigation strategies such as wire withdrawal, rotation (counterclockwise or clockwise) of the delivery system, and modulation of device flexion in select cases may help reduce noncoaxial deployment but will need to be studied further.

This study identified redeployment noncoaxial TAVR valve position, RAO, and caudal fluoroscopic coplanar angles as predictors of noncoaxial TAVR placement. Further research is needed to examine the clinical implications of noncoaxial valve deployment and develop appropriate mitigation strategies.

## Ethics Statement

This study adhered to all institutional and relevant ethical guidelines.

## Funding

The authors have no funding to report.

## Disclosure Statement

Houman Khalili reports receiving research grant and consulting fees from Edwards Lifesciences.

The other authors had no conflicts to declare.
